# Hyaluronic Acid-Chitosan Nanoparticles to Deliver Gd-DTPA for MR Cancer Imaging

**DOI:** 10.3390/nano5031379

**Published:** 2015-08-20

**Authors:** Li Zhang, Tingxian Liu, Yanan Xiao, Dexin Yu, Na Zhang

**Affiliations:** 1 Department of Pharmaceutics, School of Pharmaceutical Science, Shandong University, Ji’nan 250012, China; E-Mails: zl3553@163.com (L.Z.); liutx901115@163.com (T.L.); xiaoyn910424@163.com (Y.X.); 2 Department of Radiology Medicine, Affiliated Qilu Hospital, Shandong University, Ji’nan 250012, China; E-Mail: ydx0330@sina.com

**Keywords:** tumor imaging, magnetic resonance imaging, hyaluronic acid, chitosan nanoparticles, Gd-DTPA

## Abstract

Molecular imaging is essential to increase the sensitivity and selectivity of cancer diagnosis especially at the early stage of tumors. Recently, polyionic nanocomplexes (PICs), which are composed of polyanions and opposite polycations, have been demonstrated to be a promising strategy for biomedical applications. In this work, chitosan-hyaluronic acid nanoparticles (GCHN) were developed to deliver Gd-DTPA as MRI contrast agents for tumor diagnosis. The Gd-labeled conjugates (CS-DTPA-Gd) were successfully synthesized by carbodiimide reaction, and then GCHN were prepared by ionic gelation using the obtained CS-DTPA-Gd and hyaluronic acid. The morphology of GCHN was spherical or ellipsoidal, which is observed by transmission electronic microscopy (TEM). The mean particle size and zeta potential of GCHN were 213.8 ± 2.6 nm and 19.92 ± 1.69 mV, respectively. The significant enhancement of signal intensity induced by GCHN was observed both *in vitro* and *in vivo*. Also, compared with Magnevist, GCHN was witnessed for a prolonged imaging time in the B16 tumor-bearing mice model. Furthermore, GCHN were verified as below toxic both *in vitro* and *in vivo*. These results indicated that GCHN could potentially be an alternative to current MRI contrast agents for tumor diagnosis.

## 1. Introduction

Cancer is a major public health problem with high incidence and mortality worldwide. Accurate diagnosis of premalignant and malignant lesions at the early stage is critical for improving the currently poor survival of cancer patients in the clinic [1,2]. Despite significant progress in conventional chemotherapy, the early diagnosis of tumor poses a tremendous challenge to effective cancer therapy. An urgent need still exists for constructing excellent strategies that are capable of early diagnosis of cancer.

Magnetic resonance imaging (MRI) has become a powerful imaging modality for cancer diagnosis clinically since its application in the 1970s. Compared with other imaging tools, MRI has the advantages of non-invasiveness, no ionizing radiation, infinite penetration depth, precise three-dimensional positioning ability and high spatial resolution, especially for soft tissues [3,4]. Contrast agents (CAs) are used in most MRI diagnosis to enhance signal intensity and improve the contrast between magnetically similar but histologically distinct tissues [5].The most extensively currently used CAs in the clinic are paramagnetic gadolinium (Gd) chelates, such as Gd-DTPA (Magnevist^®^, Schering AG, Berlin, Germany), Gd-DOTA (Dotarem^®^, Guerbet, Aulnaysous-Bois, France), Gd-HP-DO3A (ProHance^®^, Bracco Diagnostics, Princeton, USA), Gd-BOPTA (Multihance^®^, Bracco Diagnostics, Princeton, USA), Gd-DTPA-BMA (Omniscan®, Amersham, Princeton, USA) and so on. Gd-chelates could increase the signal intensity by decreasing the longitudinal relaxation time (T1) of surrounding water protons [6]. Gd ions have large magnetic moment because of its seven unpaired electrons, and relatively long electronspin-relaxtion time (10^−9^ s) in the magnetic field. Therefore, Gd ions can provide high relaxivity than other paramagnetic metal ions. A total coordination number of nine enables Gd to easily form stable chelates, avoiding high toxicity in free form with polyaminocarboxylate ligands such as diethylenetriaminepentaacetic acid (DTPA), 1,4,7,10-tetraazacyclododecane-*N*,*N*',*N*'',*N*'''-tetraacetic acid (DOTA) and others [7].

Unfortunately, despite these promising properties of Gd-chelates, the biomedical applications are hindered by intrinsic low efficiency, short imaging time *in vivo* (because of fast renal clearance resulting from low molecular weight) and non-specificity to target organs [8]. Thus, creating an ideal Gd-based contrast agent with non-toxicity, higher imaging ability, specific distribution, and prolonged imaging time *in vivo* becomes a great challenge. A number of nanocarriers have been designed for Gd-based chelates delivery and have shown great potential such as enhanced contrast, prolonged imaging time, targeting properties by the enhanced permeability and retention (EPR) effect, and low toxicity. Polymeric nanoparticles [9], gold and inorganic nanoparticles [10,11], macromolecules [5,12], liposomes [13], micelles [14], dendrimers [15], are included.

Among them, polyionic nanocomplexes (PICs), which are composed of polyanions and opposite polycations, have been demonstrated to be a promising strategy for biomedical applications, which are characterized with good biocompatibility, controlled drug release, and low toxicity [16,17]. Moreover, the preparation process of PICs has the advantages of simplicity, and versatility, and avoiding the use of organic solvents. Among numerous PIC systems, chitosan-hyaluronic acid nanoparticles have been broadly explored for drug delivery [18]. Chitosan (CS) is a linear polycationic polysaccharide, which is composed of *N*-acetyl-d-glucosamine and d-glucosamine units, with one amino group and two hydroxyl groups in the repeating glucosidic residue. It is produced by the deacetylation of chitin, which is the second most abundant polysaccharide in nature and found in the exoskeleton of crustacea, insects, and some fungi. CS has been widely used in the development of nanocarriers owing to its biocompatibility, non-toxicity, low immunogenicity, and biodegradability [19]. Also, the gel-forming capability, high adsorption capacity, and inherent pharmacological properties including antibacterial, antifungal, and antitumor activity contributed to its application [20]. Furthermore, CS can transiently open the tight junctions between epithelial cells, thus facilitating drug transport across cellular barriers [21]. Hyaluronic acid (HA), on the other hand, is an anionic polysaccharide of *N*-acetyl-d-glucosamine and d-glucuronic acid with excellent biocompatibility and biodegradability. It is naturally found in the extracellular matrix and synovial fluids of the human body [22]. HA is endowed with a tumor-targeting property through specially binding to CD44 molecule, an integral membrane glycoprotein over-expressed on the surface of various cancer cells including breast, stomach, colon, ovarian, and epithelial cancers [23,24]. Alonso *et al.* used CS and HA to produce mucoadhesive nanocarriers loaded with heparin for pulmonary delivery [25]. The resulting nanomaterials were characterized with a high drug loading efficiency (approximately 70%) and a high stability in phosphate-buffered saline (PBS) for at least 24 h. Manuel Alatorre-Meda and Carmen Remunán-López *et al.* prepared CS–HA nanoparticles for gene silencing [26]. Inclusion of HA to CS NPs resulted in a comparable silencing activity with Lipofectamine™ RNAiMAX. Also, significantly improved cell viability was observed comparing with CS nanoparticles. The isothermal titration calorimetry experiment witnessed that HA could compete with siRNA in CS binding lowering CS-siRNA binding strength by 25%, thus the release behavior of genes was promoted. Sheng and Wu *et al.* prepared HA-CS nanoparticles via the electrostatic interaction for co-delivery of MiR-34a and doxorubicin against triple negative breast cancer [27]. Efficient encapsulation for MiR-34a and doxorubicin were obtained in the resulting nanoparticles. Also, synergistic effects on tumor suppression were achieved by the co-delivery strategy. The successful application of HA-CS nanoparticles in drug delivery makes us wonder how it would be in the delivery of other agents, for example, imaging agents. However, HA-CS nanoparticles have not been studied to deliver CAs for MR imaging according to our maximum literature search range.

**Scheme 1:**
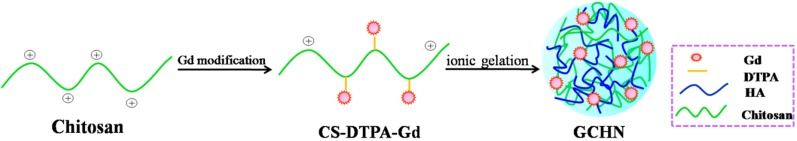
Schematic illustration of the construction of GCHN.

The aim of this study is to prepare HA-CS nanoparticles delivering Gd-DTPA as MRI contrast agents for tumor diagnosis. Gd-loaded strategy is an important factor in the construction of efficient molecular MRI contrast agents. Gd-chelates can be encapsulated into the core, or modified on the surface of nanocarriers, or conjugated with polymers. The relaxivity of Gd-based contrast agents is partly dependent on the number of Gd per nanocarrier and their exchange rate with surrounding water protons, so it is necessary to increase the number of Gd per nanocarrier and load more Gd on the surface of nanocarriers [28]. Compared with physical encapsulation, chemical connection can modify Gd ions on the surface of nanocarriers. The Gd loading content can be easy to adjust by feed ratio in synthesis. Furthermore, for physical encapsulation such as electrostatic interaction, the possibility of competition by other polyelectrolytes *in vivo* may lead to a release of the loaded Gd. Chemical conjugation can avoid this competition. In the present work, Gd-DTPA conjugated CS (CS-DTPA-Gd) was synthesized by carbodiimide reaction, and then the Gd-labeled CS and HA was employed to prepare nanoparticles (GCHN) by ionic gelation method with sodium tripolyphosphate (Scheme 1). The particle size, zeta potential, and morphology of GCHN were characterized. The MRI imaging ability of GCHN both *in vitro* and *in vivo* was evaluated, respectively. The cytotoxicity of GCHN was tested using MTT method in B16, HepG2, and A549 cells. Moreover, the *in vivo* safety of GCHN was investigated by histological assessments.

## 2. Results and Discussion

### 2.1. Synthesis of CS-DTPA-Gd

In the published work [29], Gd was modified on the surface of chitosan nanoparticles by post modification. The relaxivity of Gd-based contrast agents is partly dependent on the number of Gd per nanocarrier and their exchange rate with surrounding water protons [5]. Although this approach can effectively increase the relaxation rate, the post-modification may also affect the characteristics of the nanoparticles. Moreover, chitosan nanoparticles were obtained by cross-link between amino group and phosphate group. And Gd was modified by the reaction between amino group of chitosan and carboxyl group of DTPA. The competitiveness may result in low Gd loading. Thus, in the present work, CS-DTPA-Gd was synthesized firstly by carbodiimide reaction. It is easy to control the Gd loading by adjusting the feed ratio during the synthesis.

**Figure 1:**
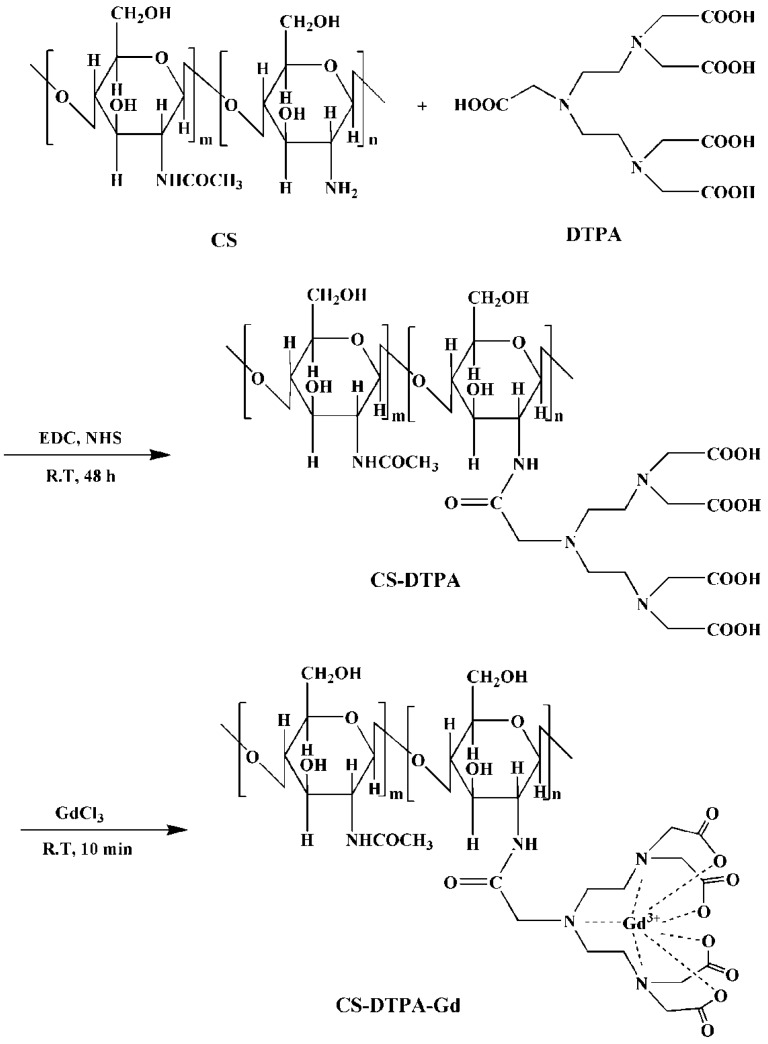
Synthesis scheme of CS-DTPA-Gd.

**Figure 2:**
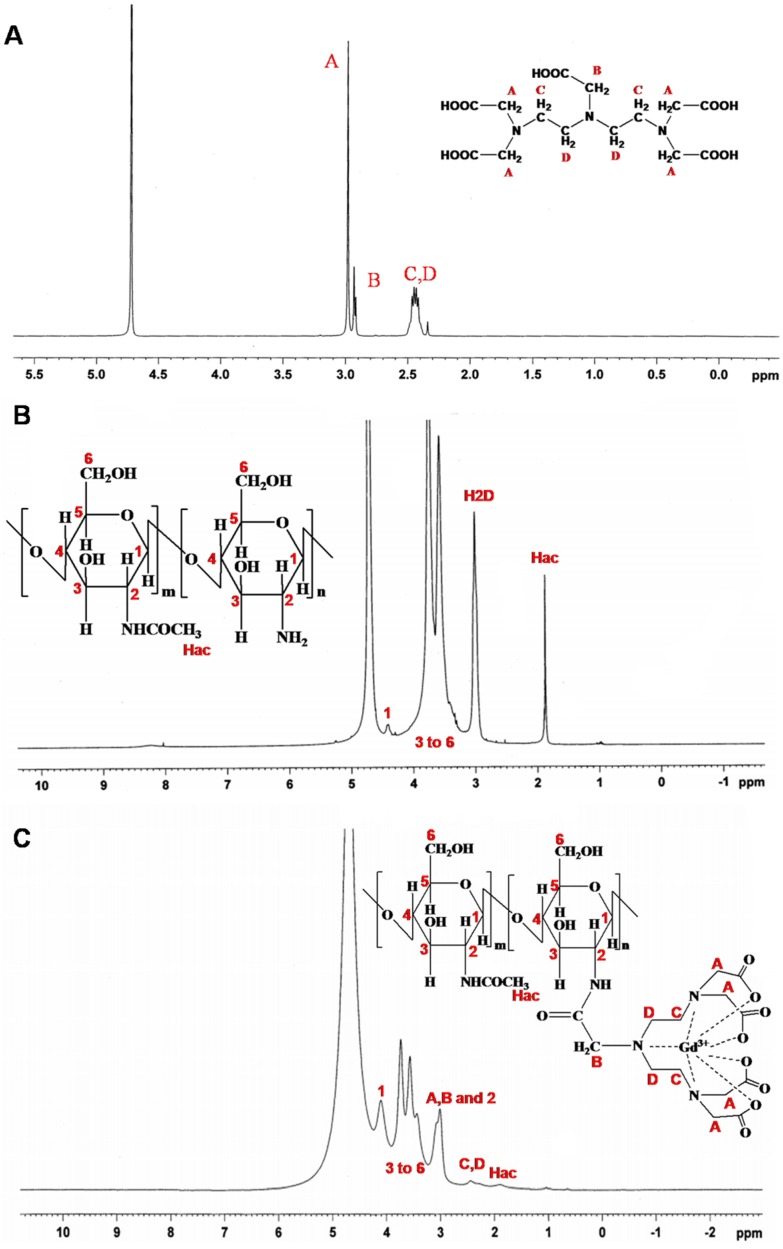
^1^HNMR spectra of DTPA (**A**); CS (**B**) and CS-DTPA-Gd (**C**).

**Figure 3:**
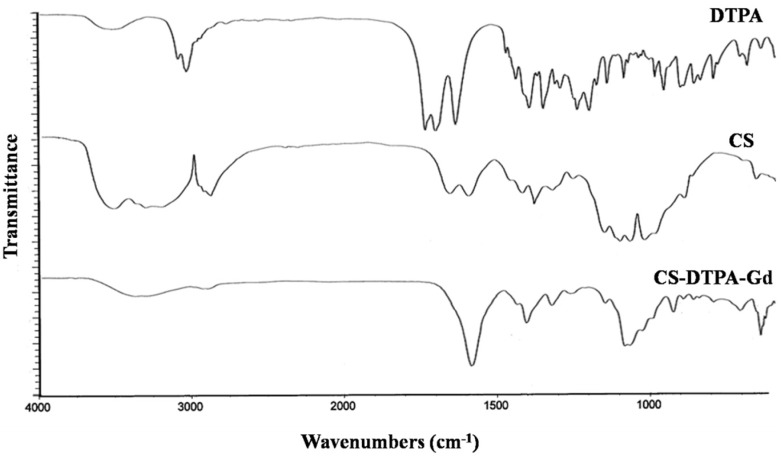
FTIR spectra of DTPA, CS and CS-DTPA-Gd.

The CS-DTPA-Gd conjugate was performed using EDC/NHS as couple agent, which was shown in Figure 1. ^1^H NMR and FTIR was applied to confirm the product structure, and the spectra were shown in Figure 2 and Figure 3, respectively. In the spectrum of CS-DTPA-Gd, the peaks at 3.0–4.5 ppm were attributed to the protons of C1, C3–6, and the peaks at 2.0 ppm were attributed to the protons of non-deacetylated monosaccharide units. The peaks at 2.0–3.0 ppm were attributed to an overlap of the proton binding the C2 of CS and the protons of DTPA. The grafting of DTPA to CS is induced by the conjugation of carboxyl groups and amino groups, which resulted in the formation of an amide bond which can also be confirmed in FTIR spectrum, as shown in Figure 3. For DTPA, the peaks at 1732, 1700 and 1634 cm^−1^ were corresponding to the C=O bending of COOH in the form of monomer, dimer, and carboxylate, respectively. For CS, the peaks at 1658 and 1597 cm^−1^ were attributed to amides I and II, respectively. The large band between 1100 and 1000 cm^−1^ was due to the vibration modes such as the stretching of C–O–C and C–O bonds [30]. For CS-DTPA-Gd, the newly emerged strong broad band at 1589 cm^−1^ was attributed to the amides II, which resulted from the grafting of DTPA to CS [31]. Its presence suggests that DTPA is successfully attached to the CS back bone by an amide bond.

In the synthesis of CS-DTPA-Gd, different molar ratio of DTPA was added as shown in Table 1. The Gd content of CS-DTPA-Gd was determined by inductively coupled plasma emission spectrometry. As the results showed, the Gd content of the final product could not increase much more when the molar ratio of [DTPA]/[NH_2_] of CS was 0.4. In order to retain relatively more amino groups of CS for electrostatic interactions in the preparation of nanoparticles with HA, the molar ratio of [DTPA]/[NH_2_] was selected as 0.4 for the remaining study.

**Table 1:** DTPA feed ratio and the final concentration of gadolinium.

Name	CS-DTPA-Gd20	CS-DTPA-Gd40	CS-DTPA-Gd60
[DTPA]/[NH_2_]	0.2	0.4	0.6
*n*_NH2_ (mmol)	0.3	0.3	0.3
*n*_DTPA_ (mmol)	0.06	0.12	0.18
*n*_EDC_ (mmol)	0.39	0.78	1.17
*n*_NHS_ (mmol)	0.39	0.78	1.17
*m*_Gd_ (mg)	5.7	10.4	10.6

### 2.2. Preparation and Characterization of GCHN

**Figure 4:**
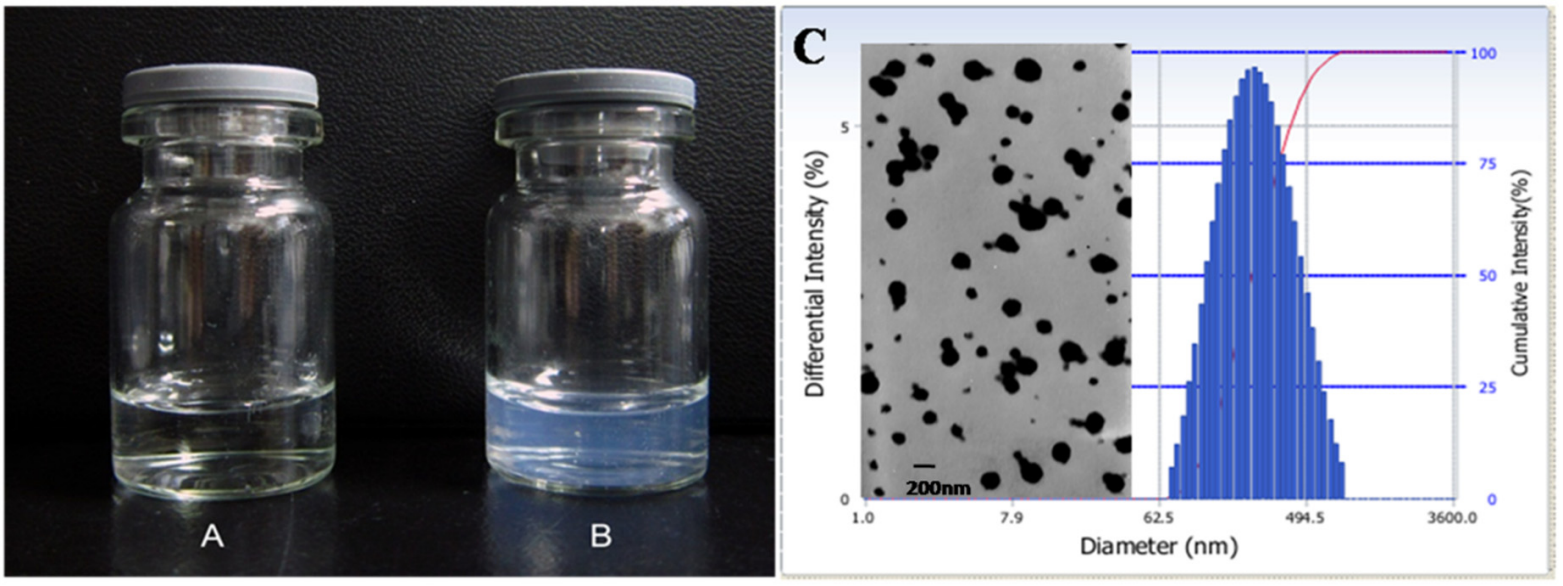
Appearance of CS-DTPA-Gd (**A**) and GCHN (**B**), TEM image and particle size distribution of GCHN (**C**).

Since the ionic cross-linking process in preparation of GCHN is based on electrostatic interaction, charge ratio is one key factor which affects the formation of nanoparticles significantly [32,33]. Thus, various ratios of CS-DTPA-Gd to HA were explored during the preparation of GCHN. When the ratio was low, the TEM results showed the particles were poorly formed; when the ratio was too high, the particle size increased and there was flocculent precipitate in the prepared process. Finally, the optimal ratio was determined as 100:15. The appearance of CS-DTPA-Gd and GCHN was shown in Figure 4A,B. Compared with the clear and transparent solution of CS-DTPA-Gd, the GCHN solution was observed with pale blue opalescence after ionic cross-linking. The TEM image and particle size distribution of GCHN were shown in Figure 4C. GCHN were well dispersed with spherical or ellipsoidal shapes (with PDI of 0.219). The average particle size and zeta potential of GCHN were 213.8 ± 2.6 nm and 19.92 ± 1.69 mV, respectively.

### 2.3. In Vitro Cytotoxicity

**Figure 5:**
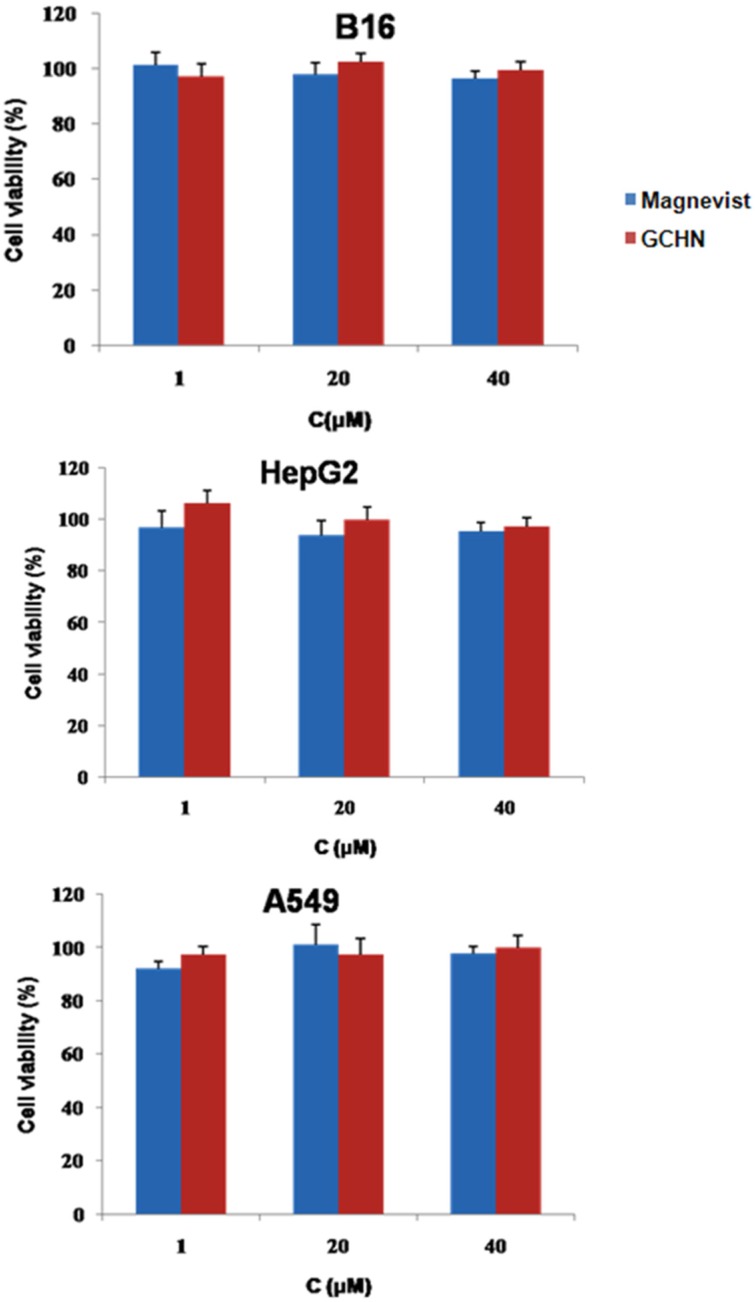
*In vitro* cytotoxicity results of Magnevist and GCHN.

MTT assays on B16, HepG2, and A549 cells were applied to evaluate the cytotoxicity of GCHN *in vitro* with different Gd concentrations. Magnevist of the same Gd concentrations was taken as a control. As shown in Figure 5, the cell viabilities of B16, HepG2, and A549 cells were all around 90% after treated with GCHN, implying that there is no obvious cytotoxicity of GCHN. Also, there is no significant difference of cell viability between GCHN and commercial products Magnevist (*p* > 0.05). This at least indicated that the cytotoxicity of GCHN will not rise when compared with Magnevist at the same concentrations. These results showed that GCHN were low toxicity and safe against the B16, HepG2, and A549 cells at the test concentrations. This was in accordance with the non-toxicity, low immunogenicity, good biocompatibility and biodegradability of both CS and HA.

### 2.4. MRI In Vitro

**Figure 6:**
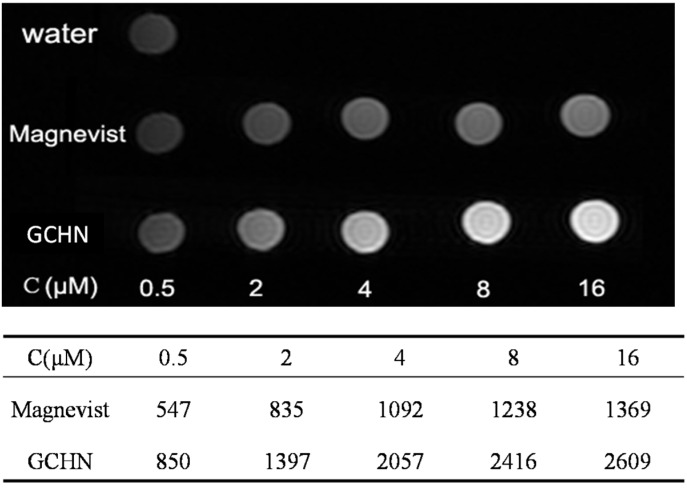
MRI images and the signal intensity *in vitro* of Magnevist and GCHN.

To explore the potential of GCHN as MRI contrast agents, GCHN and Magnevist at different Gd concentration (0.5, 2, 4, 6, 8, 16 μM) were evaluated by 3.0 T clinical MR scanner (GE, Milwaukee, WI, USA) at 25°C. For T1-weighted imaging, the higher the Gd concentration is, the higher signal intensity it will have. The T1-weighted images and quantitative signal intensity of GCHN and Magnevist samples with different Gd concentrations were shown in Figure 6. The signal intensity of the GCHN with Gd concentration at 2 μM was comparable with the Magnevist with that of 16 μM. Compared with Magnevist, of the same, the signal intensity of GCHN were much higher with Gd concentration at 0.5 and up to 16 μM. It is suggested that GCHN had higher efficient imaging ability than Magnevist at the same Gd concentration, which could increase the sensitivity of the MRI and thereby encourage early diagnosis of the tumor. These results of MRI *in vitro* indicated that GCHN had more potential than Magnevist as an efficient MRI contrast agent. According to the relaxation theory, the conjugation of MRI contrast agents to a macromolecule can efficiently retard the rotational motion of the complex and increase the rotational tumbling time (τ*_R_*) and thereby the effective correlation time (τ*_C_*), substantially increasing relaxation rate (*r*_1_) [5]; higher relaxation rate is also dependent on the rapid exchange of water protons with contrast agents [28]. For GCHN, the Gd-DTPA was linked with the macromolecule CS first and then prepared into nanoparticles by ionic cross-linking method. Since the preparation of GCHN was based on the electrostatic interaction between the free amino groups of CS and the polyanions of TPP, the modified Gd-DTPA would mainly distribute on the surface of nanoparticles, which was better for the exchange of Gd with water protons. Therefore, GCHN could significantly enhance both the relaxivity and the imaging signal intensity of Gd-DTPA.

### 2.5. MRI In Vivo

**Figure 7:**
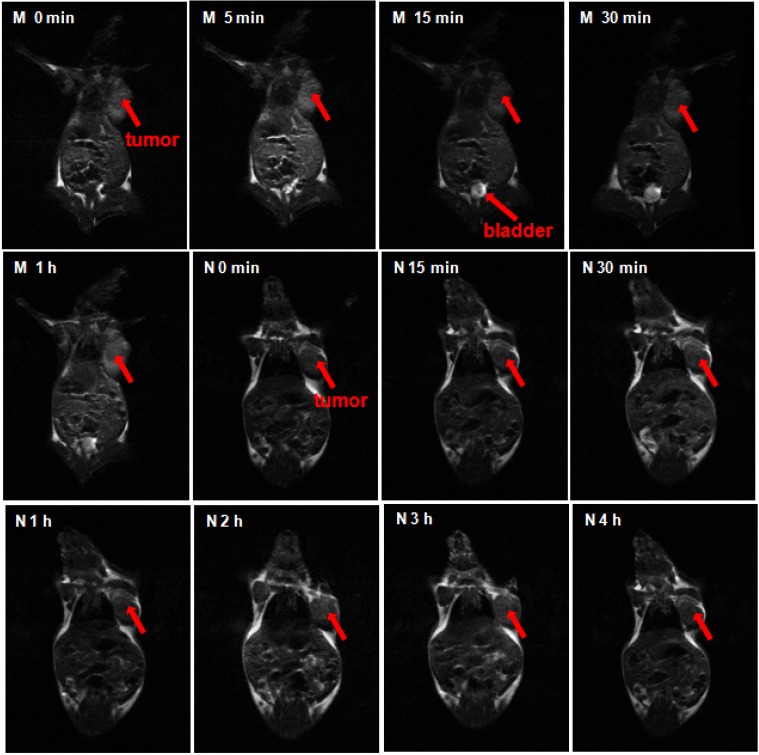
The MRI maps of mice after injected Magnevist (M) and GCHN (N).

**Figure 8:**
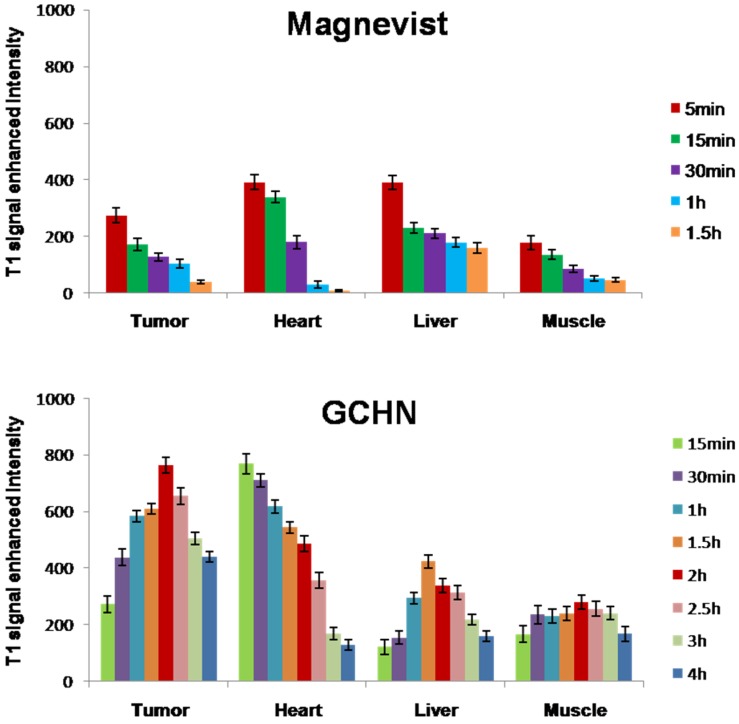
The results of the enhanced signal intensity in different tissue *in vivo*.

Tumor-bearing mice were administrated by the tail vein injection with GCHN and Magnevist at the dose of 40μmolGd/kg, respectively. To examine whether the HA-positive cells’ targeting effect could be induced by GCHN *in vivo*, a subcutaneous B16 cell xenograft model was selected as the tumor model. As reported by literature, CD44, the specific receptor of HA, was overexpressed on B16 cells [34,35]. The CD44 expression level of B16 cells used in this study was tested to be 81.18%, which was conducted by FITC labeled anti-human/mouse CD44 using flow cytometry. The MRI maps of mice and the enhanced signal intensity in different tissue *in vivo* after injected with Magnevist and GCHN were shown in Figure 7 and Figure 8, respectively.

The whole body of the mice was gray as well as the tumor before injection. After the administration of the Magnevist, the small molecular contrast agents distributed non-specifically all over the body in a very short time. The signal intensity reached to the maximum in 5 min, then following with a rapid decline. The signal intensity of the tumor increased to the maximum 273.82 ± 25.41, and the enhanced intensity of liver and heart were 389.83 ± 24.50 and 390.88 ± 26.17, respectively. The bladder began to become bright at 15 min as shown in Figure 8 (M 15 min). This was because the Gd-DTPA was eliminated rapidly by renal function. After injection for 1 h, the signal intensity of the whole mouse body became normal (Figure 8, M 1 h). After the injection of GCHN, the signal intensity gradually increased to the maximum after 2 h along with the distribution and accumulation of nanoparticles *in vivo*. There was a prolonged imaging time with GCHN injection. Moreover, the enhanced intensity of tumor and liver were 763.58 ± 28.91 and 337.79 ± 24.16, respectively. Compared with the Magnevist group, the imaging signal intensity in tumor was enhanced significantly. And the slightly reduced imaging signal intensity in the liver may be caused in part by the targeting effect of GCHN. The signal intensity began to decline slowly and certain enhanced intensity was still observed after 4 h. The prolonged imaging effect was also obvious.

### 2.6. Histological Assessment

**Figure 9:**
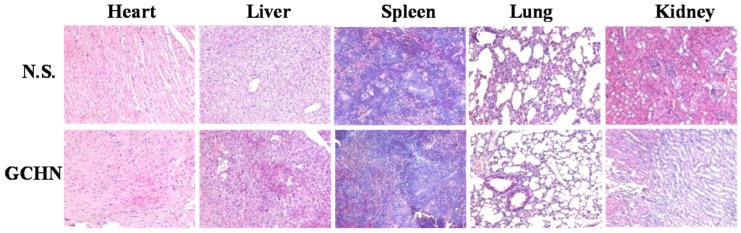
Histological assessment results (200×) for control and GCHN. No signs of tissue damage, inflammation, or lesions were observed in representative organs.

A histological analysis of organs (heart, liver, spleen, lung, and kidney) was performed to determine whether or not GCHN could cause tissue damage, inflammation, and lesions. GCHN and physiological saline were injected into Kunming mice by the tail vein. A week later, all animals were sacrificed, the liver, heart, spleen, lung, and kidney were separated, washed twice with PBS and fixed in 4% formaldehyde for histological examination. As shown in Figure 9, compared with the control group, no visible difference was observed histologically for representative organs of GCHN group. These results indicated that GCHN displayed good safety *in vivo*. GCHN were prepared by CS and HA, which both were natural polysaccharides with the advantages of non-toxicity, low immunogenicity, good biocompatibility, and biodegradability. The results were in accordance with content in the reported literatures.

## 3. Experimental Section

### 3.1. Materials

Chitosan (MW 20 kDa, degree of deacetylation (DDA) 90%) was obtained from Haidebei Bio-Engineering Co. Ltd. (Ji’nan, China).Hyaluronic acid (MW 79 kDa) was provided by Freda Biochem Co Ltd. (Ji’nan, China). Gadopentetic acid dimeglumine salt injection (Gd-DTPA, Magnevist^®^) was purchased from Bayer Schering Pharma AG (Germany). FITC labeled anti-human/mouse CD44 was purchased from eBioscience (San Diego, CA, USA). Gd_2_O_3_, DTPA and 3-(4,5-dimethylthiazol-2-yl)-2,5-diphenyltetrazoliumbromide (MTT) were purchased from Sigma-Aldrich (USA). *N*-hydroxysuccinimide (NHS), *N*-(3-dimethylaminopropyl)-*N*'ethylcarbodiimidehydrochloride (EDC), and *N,N,N',N'*-tetramethylethylenediamine (TEMED) were obtained from Aladdin (Shanghai, China). All other reagents of analytical grade were obtained commercially.

Murine malignant melanoma cells (B16), human hepatocellular liver carcinoma cells (HepG2), and human lung carcinoma cells (A549) were provided by Shandong Institute of Immunopharmacology and Immunotherapy (Jinan, China).

Kunming mice (20 ± 2 g) were obtained from the Experimental Animal Center of Shandong University. Animal experiments were carried out according to the requirements of the Animal Management Rules of the Ministry of Health (China).

### 3.2. Synthesis of CS-DTPA-Gd

CS-DTPA-Gd was synthesized by carbodiimide reaction with EDC and NHS as catalysis (Figure 1) [36]. CS (50 mg, *n*_NH2_ = 0.3 mmol) was dissolved in 20 mL 1% (*v*/*v*) acetic acid solution and the pH was adjusted to 4.7 with 1 M NaOH. 47 mg DTPA ([DTPA]/[NH_2_] = 0.4), which was pre-activated with a mixture of EDC ([EDC]/[COOH] = 1.3) and NHS ([NHS]/[COOH] = 1.3) in TEMED/HCl buffer solution (pH 4.7), was added to the CS solution. Then the solution was stirred for 72 h at room temperature. The DTPA modified CS (CS-DTPA) was obtained.

Gd ions were chelated to the DTPA groups of CS-DTPA according to the process published previously by our group [37]. Sufficient amount of GdCl_3_ (50 mg), which was obtained from Gd_2_O_3_ and HCl, was added dropwise into CS-DTPA. Then the mixture solution was stirred for another 10 min for complete conjugation between Gd ions with carboxyl groups of DTPA. The resulting product (CS-DTPA-Gd) was purified using a dialysis tube (3500 MWCO) against distilled water for three days, with water being replaced every 5h, and finally followed with lyophilization. The Gd content of CS-DTPA-Gd was determined by inductively coupled plasma emission spectrometry (IRISAdvantage, Thermo Electron Corporation, USA). Structure of the product was characterized by nuclear magnetic resonance spectroscopy (^1^H NMR) and Fourier transforms infrared spectroscopy (FTIR).

### 3.3. Preparation of GCHN

GCHN was prepared by ionic gelation using the obtained CS-DTPA-Gd and HA according to the method reported with some modification [32,38]. The pH value of CS-DTPA-Gd solution (2 mL, 2 mg/mL) was adjusted to 4. The HA solution (1 mg/mL) was mixed with cross-linker TPP (2 mg/mL, 300 μL) first. The resulting solution was added rapidly to the CS-DTPA-Gd solution under vortex mixing, following with 30 min incubationat room temperature for complete complexation. The mass ratio of CS-DTPA-Gd and HA was 100:15.

### 3.4. Characterization of GCHN

The morphology of GCHN was observed by transmission electronic microscopy (TEM) (JEM-1200EX, Jeol, Tokyo, Japan). A droplet of nanoparticles suspension was placed on a copper grid, standing for one minute. Then the excess liquid was removed by a small piece of filter paper, following with negative staining with one drop of 2% phosphotungstic acid solution for contrast enhancement. The air-dried samples were then directly observed by TEM.

The mean particle size and surface zeta potential of GCHN were measured by photon correlation spectroscopy (PCS) with Delsa™ nano C particle analyzer (Beckman Coulter, Fullerton, USA). All analysis was repeated in triplicate and the results were represented as mean ± SD (*n* = 3).

### 3.5. Cell Culture

B16 were cultured in Roswell Park Memorial Institute (RPMI) 1640 medium supplemented with 10% FBS, streptomycin at 100 mg/mL and penicillin at 100 U/mL. HepG2 and A549 were cultured in Dulbecco’s Modified Eagle Medium (DMEM) supplemented with 10% FBS, streptomycin at 100 mg/mL and penicillin at 100 U/mL. All cells were cultured in a 37° Cincubator with 5% CO_2_.

### 3.6. In Vitro Cytotoxicity

MTT assays were carried out on B16, HepG2, and A549 cells to investigate cytotoxicity of GCHN *in vitro* [9]. The cells were seeded in a 96-well plate with a density of 4000 cells/well of B16, and 8000 cells/well of HepG2 and A549, respectively. After overnight incubation for cell attachment, the medium was replaced with GCHN and Magnevist at Gd concentrations of 1, 20, and 40 μM. After 24 h incubation, 20 μL MTT solution (5mg/mL) was added to each well and the plate was incubated for another 4 h. The plates were centrifuged (3000 rpm, 10 min) and the media was removed. 200 μL DMSO was added to each well to fully dissolve the for mazan crystals formed by the living cells. The absorbance at 570 nm of the obtained solution was recorded by a microplate reader (Model 680, BIO-RAD, Hercules, USA). Five wells were set for each concentration, with blank wells and control wells at the same time. All the assays were repeated three times. The cell viability (%) was calculated according to the following equation:

$$\text{Cell viability }\left(\text{\%}\right)=\frac{\text{Abs}\left(\text{sample}\right)-\text{Abs}\left(\text{blank}\right)}{\text{Abs}\left(\text{control}\right)-\text{Abs}\left(\text{blank}\right)}\times 100\text{\%}$$

where Abs(sample) was the absorbance of the cells treated with tested samples, Abs(control) was the absorbance of the cells treated with culture medium, and Abs(blank) was the absorbance of culture medium without cells.

### 3.7. MRI In Vitro

To explore the potential of GCHN as MRI contrast agent, MRI *in vitro* was performed using a 3.0 T Sigma scanner (GE, Milwaukee, WI, USA) according to the method published previously by our group [39].The T1-weighted MR images of GCHN and Magnevist were obtained with different Gd concentrations (0.5, 2, 4, 6, 8, 16 μM) using Tl-weighted pulse sequences, respectively. The echo time (TE) was set as 15 ms, while the repetition time (TR) was set as 300 ms. The signal intensity of the samples was measured on the obtained T1-weighted MR images.

### 3.8. MRI In Vivo

To explore the *in vivo* imaging ability and tissue distribution of GCHN, the MRI *in vivo* test was performed on B16 tumor-bearing mice using a 3.0 T Sigma scanner (GE, Milwaukee, WI, USA) according to the method published previously by our group [39]. The CD44 expression levels of B16 cells that used in this study were detected with FITC labeled anti-human/mouse CD44 by flow cytometry. Kunming mice were implanted with B16 cells by subcutaneously injecting with 0.1 mL of B16 cell suspension at the axillary space. GCHN and Magnevist were injected into mice through the tail vein at a dose of 40 μmol Gd/kg, respectively. After about 10 days of normal breeding, when the tumor diameters were approximately 1.5 cm, the mice were imaged on a Sigma scanner using a shoulder coil. T1 dynamic scans were taken before injection and at various time points after administration (5 min, 15 min, 30 min, 1 h, 1.5 h, 2 h, 2.5 h, 3 h and 4 h). Quantitative T1 MR Images were acquired by saturation-recovery multi-slice spin-echo pulse sequence. Saturation-recovery T1 images of three or four slices with slice thickness of 2 mm were obtained with six relaxation delays of 167 ms with an inplane spatial resolution of 0.250 mm (128 × 64 matrix zerofilled to 128 × 128, number of scans = 8, field of view = 10 cm × 15 cm). The T1 signal intensity was tested by MRI workstation (ADW 4.2). T1 signal enhanced intensity was calculated according to the following equation:

T1 signal enhanced intensity (*t_i_*) = T1 signal intensity (*t_i_*) − T1 signal intensity (*t*_0_)

where T1 signal intensity (*t*_0_) was T1 signal intensity before injection, and T1 signal intensity (*t_i_*) was T1 signal intensity after injection for *t_i_*.

### 3.9. Histological Assessment

In order to evaluate the compatibility and tissue toxicity of GCHN *in vivo*, a histological observation was performed [40]. GCHN was injected into five female Kunming mice through the tail vein with a dose of 40 μmol Gd/kg, and normal saline (NS) was taken as a control reagent. A week later, all the mice were sacrificed, and the heart, liver, spleen, lung, and kidney were separated. Washed twice with PBS, all the organs were fixed in 4% formaldehyde, dehydrated in gradient alcohol, placed in xylene, embedded in paraffin and made into sections, following with hematoxylin-eosin (HE) staining for histological examination with microscope.

### 3.10. Statistical Analysis

All studies were repeated three times and measured in triplicate at least. Results were expressed as mean ± SD. Statistical significance was analyzed using Student’s *t*-test and differences between experimental groups were considered statistically significant at *p* < 0.05.

## 4. Conclusions

In this study, hyaluronic acid modified gadolinium-loaded chitosan nanoparticles, GCHN, were successfully prepared as MRI contrast agents for tumor diagnosis. The Gd-labeled conjugates, CS-DTPA-Gd, was successfully synthesized first by carbodiimide reaction, and then GCHN were prepared by ionic gelation method with CS-DTPA-Gd and hyaluronic acid. The significant enhancement of signal intensity induced by GCHN was observed both *in vitro* and *in vivo*. Also, compared with Magnevist, GCHN witnessed prolonged imaging time in B16 tumor-bearing mice model. Furthermore, GCHN were verified below toxic both *in vitro* and *in vivo*. These results indicated that GCHN could be a potential alternative to current MRI contrast agents for tumor diagnosis.
